# Targeting phosphoglycerate kinase 1 with terazosin improves motor neuron phenotypes in multiple models of amyotrophic lateral sclerosis

**DOI:** 10.1016/j.ebiom.2022.104202

**Published:** 2022-08-11

**Authors:** Helena Chaytow, Emily Carroll, David Gordon, Yu-Ting Huang, Dinja van der Hoorn, Hannah Louise Smith, Thomas Becker, Catherina Gwynne Becker, Kiterie Maud Edwige Faller, Kevin Talbot, Thomas Henry Gillingwater

**Affiliations:** a Edinburgh Medical School: Biomedical Sciences, University of Edinburgh; Edinburgh, UK; b Euan MacDonald Centre for Motor Neuron Disease Research; Edinburgh, UK; c Nuffield Department of Clinical Neurosciences, University of Oxford; Oxford, UK; d Center for Regenerative Therapies at the TU Dresden, Technische Universität Dresden, Dresden, Germany; e Royal (Dick) School of Veterinary Studies, University of Edinburgh; Edinburgh, UK

**Keywords:** Motor neuron disease (MND), Bioenergetics, Drug repurposing, Neuroprotection

## Abstract

**Background:**

Amyotrophic lateral sclerosis (ALS) is a fatal neurodegenerative disorder with heterogeneous aetiology and a complex genetic background. Effective therapies are therefore likely to act on convergent pathways such as dysregulated energy metabolism, linked to multiple neurodegenerative diseases including ALS.

**Methods:**

Activity of the glycolysis enzyme phosphoglycerate kinase 1 (PGK1) was increased genetically or pharmacologically using terazosin in zebrafish, mouse and ESC-derived motor neuron models of ALS. Multiple disease phenotypes were assessed to determine the therapeutic potential of this approach, including axon growth and motor behaviour, survival and cell death following oxidative stress.

**Findings:**

We have found that targeting a single bioenergetic protein, PGK1, modulates motor neuron vulnerability *in vivo*. In zebrafish models of ALS, overexpression of PGK1 rescued motor axon phenotypes and improved motor behaviour. Treatment with terazosin, an FDA-approved compound with a known non-canonical action of increasing PGK1 activity, also improved these phenotypes. Terazosin treatment extended survival, improved motor phenotypes and increased motor neuron number in Thy1-hTDP-43 mice. In ESC-derived motor neurons expressing TDP-43^M337V^, terazosin protected against oxidative stress-induced cell death and increased basal glycolysis rates, while rescuing stress granule assembly.

**Interpretation:**

Our data demonstrate that terazosin protects motor neurons via multiple pathways, including upregulating glycolysis and rescuing stress granule formation. Repurposing terazosin therefore has the potential to increase the limited therapeutic options across all forms of ALS, irrespective of disease cause.

**Funding:**

This work was supported by project grant funding from MND Scotland, the My Name’5 Doddie Foundation, Medical Research Council Doctoral Student Training Fellowship [Ref: BST0010Z] and Academy of Medical Sciences grant [SGL023\1100].

**Research in context**

**Evidence before this study**

Amyotrophic lateral sclerosis (ALS) is a devastating, fatal neurodegenerative disease, characterised by the specific cell death of motor neurons and without effective treatment. As the majority of patients do not have a known genetic cause, therapy development has been challenging. Dysregulated energy metabolism is common across both familial and sporadic forms of ALS, and so targeting energy production has the potential to be therapeutic for multiple patient groups. The FDA-approved compound terazosin has previously been identified as targeting the glycolysis enzyme phosphoglycerate kinase 1 (PGK1) and increasing its activity. Terazosin was shown to be neuroprotective in models of stroke and Parkinson's disease. We therefore asked whether this neuroprotective effect could translate to ALS, a disease where novel therapeutic approaches are desperately needed.

**Added value of this study**

Here we provide pre-clinical evidence that targeting the PGK1 enzyme either genetically or using terazosin, an FDA-approved drug with a known safety profile, is neuroprotective across multiple models of ALS. Treatment with terazosin improved motor neuron phenotypes in zebrafish models which correlated with improved motor behaviour; it also increased survival and clinical phenotypes in a mouse model of ALS and protected against cell death in response to oxidative stress in motor neurons in culture. The neuroprotective action of terazosin is likely due to the observed increase in glycolysis, as well as potentially through a recovery of stress granule formation seen in motor neuron cultures.

**Implications of all the available evidence**

From these data we conclude that terazosin is a promising candidate for clinical trials in ALS. Since terazosin is acting on the glycolysis pathway and therefore downstream of the cause of disease, it has the potential to benefit patients with all forms of ALS, irrespective of disease cause. Finally, by repurposing an FDA-approved drug, this could result in a faster translation to bedside.

Alt-text: Unlabelled box

## Introduction

Amyotrophic lateral sclerosis (ALS) is the most common form of motor neuron disease, with a lifetime risk of around 1:350 for men and 1:400 for women,^1^ and the incidence varying between 2.1 and 3.8 per 100,000 depending on the population studied.^2^ This devastating disease, characterised by the loss of upper and motor neurons leading to progressive weakness, is uniformly fatal: 50% of patients die within 3 years from first symptoms and 80-90% within 5 years. Crucially, there are currently no treatment options for patients that meaningfully alter the disease course, with the only approved drugs, riluzole and edavarone, increasing lifespan by a few months.^3^^,^^4^

A major obstacle to therapy development for ALS is the heterogeneity of the disease, with the discovery of dozens of disease-associated genetic mutations leading to dysregulation of multiple molecular pathways. The majority of ALS cases are apparently sporadic, and while some of these patients do carry ALS-causing genetic mutations, most do not have a known genetic cause.^5^ Of the 10% familial ALS cases, the most common genetic mutation is a repeat expansion in an intronic region of the *C9ORF72* gene, which still only accounts for around a third of patients with a family history of ALS and 5% of apparently sporadic cases.^5^ Less prevalent gene mutations are found in the *SOD1* gene in 20% of familial cases*,* and in the *TARDBP* and *FUS* genes, although these account for very few ALS patients. While more, rare mutations continue to be associated with ALS,^6^ it is unlikely that there will ever be a unifying genetic explanation for ALS on which therapy can be designed. Instead, it seems that multiple genetic risk variants interact with environment and age-related stochastic change in the nervous system in a multistep pathogenic pathway.^7^ This leads to further heterogeneity in the molecular mechanisms contributing to the specific cell death of motor neurons, from impaired DNA repair and RNA processing to protein aggregation to cytoskeletal dysfunction.^8^ Despite the varied disease mechanisms, one commonality across most ALS patients is the presence of TDP-43 positive inclusions in neurons.^9^ TDP-43 is an RNA-binding protein normally shuttled between the nucleus and cytoplasm^10^ and its sequestration in the cytoplasm has been linked to multiple pathogenic mechanisms including altered RNA metabolism,^11^ impaired stress granule formation,^12^ and axonal transport.^13^

Due to this complex pathogenesis, targeting pathways known to be dysfunctional across multiple ALS models could provide benefits to a broad range of ALS patients regardless of initial cause of disease onset. Mitochondrial dysfunction, increased oxidative stress and decreased ATP production have been linked to a number of ALS-associated genes including *SOD1*,^14^ *TARDBP*,^15^ and *C9ORF72*^16^ as well as in tissue from sporadic patients.^17^^,^^18^ Bioenergetic pathways therefore appear to be a commonality across forms of ALS^19^^,^^20^ and therefore make attractive targets for therapy development. Indeed, metabolic dysfunction is seen in ALS fibroblast samples^21^ and metabolic dysfunction and weight loss is negatively associated with survival in ALS patients.^22^, ^23^, ^24^

Amongst the intricate web of metabolic pathways, evidence suggests that glycolysis may be a useful target. The key glycolytic enzyme phosphoglycerate kinase 1 (PGK1) was found to be downregulated in astrocytes from the SOD1^G93A^ mouse model,^25^ whilst overexpression of phosphofructokinase in *Drosophila* led to amelioration of TDP-43-induced locomotor defects.^26^ Glycolytic ATP production was reduced in i-motor neurons derived from both sporadic and familial ALS patients^27^ while fibroblasts from ALS patients show a 1.7-fold decrease in PGK1 expression.^28^ We have previously shown that PGK1 is downregulated in motor neurons that are susceptible to the childhood motor neuron disease spinal muscular atrophy (SMA).^29^ PGK1 activity can be increased using an off-target effect of the FDA-approved small molecule terazosin, which is normally prescribed for benign prostatic hyperplasia or hypertension.^30^ The structure of terazosin has been previously described and was modelled using co-crystallisation complexes to bind to PGK1 at an overlapping site to the ATP/ADP binding site.^30^ Terazosin was found to be neuroprotective in models of stroke, SMA and Parkinson's disease (PD).^29^, ^30^, ^31^ Indeed, patients with PD who had been prescribed terazosin, or other drugs with a similar PGK1-binding motif, had fewer hospital visits and a lower scores for both motor and non-motor symptoms.^31^ By interrogating whole-country healthcare datasets from the US and Denmark, it was further shown that a prescription for terazosin decreases the risk of developing PD.^32^ Targeting PGK1 with terazosin is therefore an attractive potential therapy for ALS. Here, we have used multiple *in vitro* and *in vivo* models of ALS in zebrafish and mice to determine the therapeutic potential and mechanism of action for terazosin.

## Methods

### ALS models of zebrafish

Two models of ALS were generated in larval zebrafish: knockdown of endogenous C9orf72 using an ATG-targeting phosphorodiamidate morpholino oligomer (MO)^33^ and overexpression of the human sequence of mutant TDP-43^G348C^ RNA.^34^ The ATG-targeting antisense oligonucleotide (ATTGTGGAGGACAGGCTGAAGACAT; 1 nl of 0.02 mM; Gene Tools) was resuspended in nuclease-free water to a 2 mM stock solution. *PGK1* and *mutTDP-43* RNA was transcribed *in vitro* from *NotI*-linearised plasmids containing the human sequences of *PGK1* and *TARDBP* with the G348C mutation using the mMESSAGE mMACHINE™ SP6 Transcription Kit (ThermoFisher), followed by lithium chloride precipitation and dilution in nuclease-free water. Fertilised eggs from zebrafish overexpressing hb9:GFP, as previously described,^35^ were microinjected at the single-cell stage with 1 nl of either 0.05 mM C9orf72 MO or 25 ng/µl *mutTDP-43* RNA and compared to uninjected within-clutch controls. Animals were randomly assigned to treatment or control groups, and within-clutch controls were used in all experiments. For motor behaviour, animal caretakers and investigators were blinded to allocated treatment groups. For PGK1 overexpression experiments, fertilised eggs were co-injected with 200 ng/µl *PGK1* RNA at the single cell stage. Injected eggs and uninjected controls were incubated at 30°C. For terazosin experiments, developing embryos were moved to fresh water at 6 hours post-fertilisation (hpf) with relevant concentrations of terazosin (Sigma) and returned to 30°C incubation. For motor axon phenotype analysis, embryos were collected at 30 hpf, dechorionated and fixed in 4% PFA for 3 hours, washed with PBS-T then stored in 70% glycerol at 4°C overnight before being deyolked and whole-mounted for microscopy. Fluorescent images were taken using the Zeiss AxioImager M2 microscope with Apotome. 6 pairs of motor neurons were imaged per fish as z-stacks, beginning with the first pair of motor neurons after the yolk sac, over the yolk extension. Z-stacks were converted to maximum intensity projections for each side of the fish. Images were blinded for analysis. Axon length was measured using the Simple Neurite Tracer plug-in for ImageJ, and the average axon length per fish was taken. For branching phenotype analysis, each axon was given a score (3=“healthy” neuron, 2=mild phenotype, 1=moderate phenotype, 0=severe phenotype; as previously described) and percentage of healthy axons per fish was plotted. For the touch evoked escape response (TEER) test, zebrafish larvae were incubated for 3 days with daily water changes and with the addition of 50 µM terazosin in the water. At 3 days post-fertilisation (dpf), larvae were placed individually in the centre of a 35mm petri dish and their movements following a light tail touch tracked using EthoVision XT 8.5 software (Noldus), recording the distance travelled. Each fish was assessed for TEER 3 times, and their average distance taken.

### hTDP-43 mouse model

The mThy1-hTDP-43 mouse model (“hTDP-43”) was purchased from Jackson Laboratory (RRID:IMSR_JAX:012836; B6;SJL-Tg(Thy1-TARDBP)4Singh/J) and maintained at the University of Edinburgh under standard conditions in a 12 hour light/dark cycle. Individual animals were counted as one experimental unit. To control for body weight differences between neonates, only litters with 6-10 pups were used. Any mouse weighing more than 3g or less than 1.5g on post-natal day 5 (P5) was removed from the study (2 mice removed). hTDP-43 mice were bred as a heterozygous cross, with homozygous transgenic offspring developing a severe ALS-like phenotype and wild-type nontransgenic littermates acting as controls. Both sexes were used in all treatment groups. Animals were randomly assigned to treatment or control groups by Microsoft Excel's random number generator. Overall, 10 homozygous hTDP-43 mice were treated with saline, 13 homozygous hTDP-43 mice treated with each dose of terazosin, 11 wild-type mice were treated with saline, 6 wild-type mice treated with 10 µg/kg terazosin and 8 wild-type mice treated with 100 µg/kg terazosin: 61 mice used in total. For survival analysis and clinical scoring, animal caretakers and investigators were blinded to allocated treatment groups. Terazosin or saline control was administered via daily intraperitoneal injection from day of birth (P1). Briefly, neonatal pups were weighed and different dilutions of terazosin used in order to keep injection volumes between 5-15 µl. Pups were gently but securely scruffed and terazosin or saline control was injected intraperitoneally using a Hamilton syringe (Fisher) and 33 gauge needle (Fisher). Pups were then immediately returned to the breeding cage. Mice were weighed daily and assessed for motor dysfunction via a clinical score (Table 1). Once a mouse developed full paralysis in both hindlimbs (clinical score of 3) it was humanely culled via cervical dislocation with confirmation of death via exsanguination.

**Table 1:** Clinical scoring for hTDP-43 mouse model. A clinical score of 3 is classified as the humane endpoint for this mouse model.

Score	Tail Suspension	Grip Test	Free movement
0	Both limbs consistently splayed outward	Mouse can grip the edge of a cup with both hind paws	Normal movement, weight supported on all limbs
1	One limb retracted towards the abdomen for more than 50% of the time	Mouse shows weakness in gripping with one hind paw	Mouse has a mild tremor or limp when walking
2	Both limbs are partially retracted towards the body for more than 50% of the time	Mouse shows weakness in gripping with both hind paws	Mouse shows severe tremor and/or limp, or the feet point away from the body during locomotion (“duck feet”)
3	Both limbs are fully retracted for more than 50% of the time	Mouse cannot grip with either hind paw	Mouse has difficulty moving forward and drags its abdomen along the ground

### Motor neuron cell counts

Mice were injected with 100 µg/kg terazosin or saline control via daily intraperitoneal injection as above. Mice were euthanised at P19 by overdose of anaesthetic and confirmed death by exsanguination. Spinal cords were flushed from the vertebral column and the lumbar section removed as identified by the lumbar enlargement. Spinal cord sections were fixed in 4% paraformaldehyde overnight, dehydrated in 30% sucrose and embedded in OCT/Sucrose for cryosectioning. Spinal cords were sectioned at 25 µm thickness. NeuroTrace 500/525 (a fluorescent Nissl stain; ThermoFisher) and DAPI staining were performed on slides containing sections from a similar level in the lumbar spinal cord. Briefly, slides were rehydrated in PBS and permeabilised with PBS + 0.1% Triton-X. A concentration of 1:200 NeuroTrace 500/525 in PBS was used to stain the slides, followed by a wash in PBS + 0.1% Triton-X and extensive washing with PBS. DAPI staining (4’,6-Diamidino-2-Phenylindole; nuclear stain; ThermoFisher) was carried out at 300 nM concentration, washed off with PBS, and the slides were mounted with Mowiol (Merck) to prevent fading. Images of both ventral horns for 2-3 spinal cord sections per mouse were captured using a Nikon A1R confocal on a 20x objective. Motor neurons were counted using the criteria of cell width above 20 µm with intense Nissl signal. Total counts were averaged per ventral horn for comparison. Staining, imaging, and analysis were performed by a researcher blinded to both the mouse genotype and treatment. Terazosin-treated groups were compared to vehicle control via t-test.

### Differentiation of mouse embryonic stem cell-derived motor neurons (mESC-MNs)

Mouse embryonic stem cell (mESC)-derived motor neurons were generated in-house from ESC lines derived from TDP-43 BAC knock-in mouse expressing either human TDP-43^WT/-^ or TDP-43^M337V/-^ (RRID:IMSR_JAX:029266) at low levels as previously described.^36^^,^^37^ Differentiation of mESCs to motor neurons was performed based on previously published protocols.^37^, ^38^, ^39^ In brief, mESCs were plated onto a bed of primary mouse embryonic fibroblasts (PMEFs) and expanded in Knockout DMEM (Invitrogen) supplemented with 15% ESC-screened foetal bovine serum (ThermoFisher), 2 mM penicillin-streptomycin-glutamine (Invitrogen), 0.01% MEM non-essential amino acids (Invitrogen), 1 ng/mL leukaemia inhibitory factor (LIF), 0.01% EmbryoMax ESC qualified nucelosides (Millipore) and 0.1 mM 2-mercaptoethanol (Invitrogen). Following 2 days of expansion, embryoid bodies (EBs) were lysed from the underlying PMEFs through treatment with 0.25% trypsin-EDTA. The resulting cell suspension containing mESCs was plated into 10 cm dishes (Corning) in ADFNK media containing 50% advanced DMEM/F-12 (Invitrogen), 50% neurobasal medium (Invitrogen), 10% knockout serum replacement (Invitrogen), 2 mM penicillin-streptomycin-glutamine (Invitrogen) and 0.1 mM 2-mercaptoethanol. Following 2 days incubation in ADFNK media, Ebs were split 1:4 into 10 cm dishes in ADFNK media supplemented with 1 µM retinoic acid (RA; Sigma) and 0.5 µM smoothened agonist (SAG; Merck). After a further 3 days, Ebs were collected and dissociated in Accumax (Sigma) and cells plated onto poly-l-ornathine (1:10 in sterile water; Sigma) and laminin (2.5 µg/mL in HBSS; Invitrogen) coated plates in ADFNK media containing RA, SAG and growth factors (10 ng/mL glia derived neurotrophic factor (GDNF), 10 ng/mL BDNF, 10 ng/mL CNTF and 10 ng/mL NT-3; Preprotech). Following a further 4 days of incubation, motor neurons were analysed via cell death assays, Seahorse assays or immunohistochemical assays as described below.

### mESC-MN survival assays

mESC-MNs were plated in laminin-coated Cellcarrier ultra 96-well black walled clear bottom plates (PerkinElmer), at a density of 1.5×10^5^ cells/cm^2^. Plates were incubated at 37°C/5% CO_2_ for 24 hours. Mature TDP-43^WT^ and TDP-43^M337V^ mESC-MNs were then treated for 24 hours with 0.625, 1.25 and 2.5µM of terazosin in mESC culture media containing 0.1% DMSO. Following drug treatment, mature mESC-MNs were stressed with 0.5 mM sodium arsenite for 1 hour at 37°C/5% CO_2_, then washed once with PBS before addition of mESC culture media containing 10 µg/ml resazurin and 0.1% DMSO. Plates were incubated at 37°C/5% CO_2_ for a further 24 h prior to reading on a Perkin Elmer plate reader at Excitation 570nm/Emission 584nm. The relative survival of TDP-43 mESC-MNs treated with terazosin was normalised against stressed cells in the absence of drug.

### Seahorse XF extracellular flux assays

Mitochondrial function and glycolysis in mESC-derived motor neurons were analysed using the Seahorse Mito Stress Test and Seahorse Glycolytic Rate Assay (Agilent) as described by the manufacturer. Briefly, mESC-derived motor neuron precursors were plated into 96 well microplates (Agilent) at a density of 6.5×10^4^ cells per well in ADFNK media supplemented with RA, SAG and growth factors for four days. To investigate the effects of terazosin, cells were incubated with terazosin at 2.5 µM 24 hours prior to the assay in media supplemented with 0.1% DMSO to allow cellular uptake of the drug. Untreated cells were also incubated in media supplemented with 0.1% DMSO to allow comparison. Following treatment cells were washed once in Seahorse XF DMEM (Agilent) containing 1 mM pyruvate, 2 mM glutamine and 10 mM glucose, then incubated with 180 µL Seahorse XF DMEM at 37°C, no CO_2_ for 45 minutes. For analysis of mitochondrial function with the Mito Stress Test, stock solutions were generated for oligomycin, FCCP and rotenone/antimycin A and drugs loaded into the appropriate ports on the microplate to generate final concentrations within the wells of 100 µM oligomycin, 100 µM FCCP and 50 µM rotenone/antimycin-A. For assessment of glycolysis, stock solutions were prepared for rotenone/antimycin A and 2-deoxy-D-glucose (2-DG), with drugs loaded into ports to generate final concentrations per well of 50 µM rotenone/antimycin A and 500 mM 2-DG. For both assays, the calibration plate containing a loaded sensor cartridge was loaded into the Seahorse XF Analyzer for calibration. Subsequently, to run the assay, the calibration plate was replaced with the cell culture microplate. Following completion of the assay, oxygen consumption rate and proton efflux rate within each well was normalised by cell number using the CyQuant Cell Proliferation Assay, as described by the manufacturer (ThermoFisher).

### Stress granule analysis

For downstream analysis of stress granules, mature ESC-MNs grown in laminin-coated plates or on coverslips were stressed with 0.5 mM sodium arsenite for 1 hr at 37°C/5% CO_2_, then fixed immediately with 4% paraformaldehyde (PFA) for 15 min at RT. Stress granules were identified by immunoreactivity of specific markers (G3BP) and quantified as a stress granule if ≥ 0.2 µm in size. ≥30 random cells were analysed from stained coverslips or plates. For all analyses, ≥3 individual differentiations of ESC-MNs per genotype were used. Motor neurons cultured and fixed in laminin-coated Cellcarrier ultra 96-well black walled T/C clear bottom plates (PerkinElmer), were blocked for 1hr at room temperature (RT) with a solution of 5% normal donkey serum (NDS) and 0.01% Triton-X-100 in phosphate buffered saline (PBS), and then incubated overnight with primary antibodies against mouse anti-G3BP (Abcam, RRID:AB_941699, 1:1000) (to identify stress granules) and goat anti-Choline acetyltransferase (ChAT; Millipore; RRID:AB_2079751; 1:500) in 1:5 diluted blocking solution. Controls for the specificity of the secondary antibodies had the equivalent amount of antibody solution added, but without any primary antibody. After washing with 0.1% Triton-X/PBS for 3×10 min, cells were then washed with PBS, and incubated for 1hr at RT with secondary antibodies Alexa Fluor 488 or Alexa Fluor 568 conjugated donkey anti-mouse or anti-goat secondary antibodies (Life Technologies, 1:1000) for one hour at RT. After washing with PBS for 3×10 min, nuclei were stained with 4’,6-Diamidino-2-Phenylindole (DAPI) for 10 min. 100 µl of PBS was added to each well, and the plates imaged on an Opera Phenix plus screening system (Perkin Elmer).

### Ethics

All zebrafish and mice used in these experiments were bred and handled in accordance with University of Edinburgh and UK Home Office regulations, project licence numbers 70/8805 and P92BB9F93.

### Statistics

All data are presented as means±SEM. N numbers are reported in figure legends. All *in vivo* data represent separate biological replicates. *In vitro* experiments were repeated over at least 3 differentiations. Sample size calculations for *in vivo* experiments were performed using the NC3Rs experimental design assistant, assuming parametric data with two-tailed pairwise comparisons.^40^ Sample sizes for changes in axon length in zebrafish were based on pilot data (mean change in axon length of 13 µm, SD = 16, α = 0.05) giving a power of 0.9 to *n* = 33. Sample sizes for zebrafish behaviour were based on pilot data (mean change in distance swum of 13.6 mm, SD=8.6, α=0.05) giving a power of 0.9 to *n* = 10. Sample sizes for mouse experiments were based on pilot data (mean change in body weight 0.6g, SD=0.45, α=0.05) giving a power of 0.8 to *n* = 10. Parametric data were tested for normality using the Shapiro-Wilk test. Branching analysis and clinical scoring were classed as nominal data and so non-parametric tests were used. Parametric data were analysed using a one-way ANOVA test with Tukey's test for multiple comparisons. When comparing to a single control group, such as terazosin treatment in zebrafish models, parametric data were analysed using a one-way ANOVA test with Dunnett's test for multiple comparisons. Due to small sample size, mESC-MN data were analysed with one-way ANOVA test with Bonferroni correction. Pairwise comparisons such as for motor neuron counts were analysed using a two-tailed student's t-test. Non-parametric data were analysed using the Kruskal-Wallis test with Dunn's multiple comparisons. Kaplan-Meier curves were compared using the log-rank Mantel-Cox test. All statistical analysis was performed using GraphPad Prism version 9.

### Role of funders

The funders had no role in the study design; collection, analysis and interpretation of data; writing of this paper; or in the decision to submit the paper for publication.

## Results

### PGK1 represents a viable therapeutic target for motor neurons in ALS

Previous experimental evidence suggests that targeting the glycolytic enzyme PGK1 may confer neuroprotection across various neurodegeneration paradigms.^29^, ^30^, ^31^ We therefore wanted to establish whether this neuroprotection could be translated to motor neurons in the context of ALS. In order to test the therapeutic potential of targeting PGK1 in ALS, we initially modelled ALS-linked mutations in zebrafish. Zebrafish are a useful vertebrate model for genetic manipulation due to their translucent bodies during the first few days post-fertilisation, large clutch size allowing for within-clutch controls, and external egg-laying allowing for injections of genetic constructs at the single cell stage.^41^ Primary motor neurons are a subclass of zebrafish motor neurons that undergo axogenesis in the first day of development, which allows us to observe the effects of ALS genetic models on neurite growth *in vivo*. These axons follow a highly stereotyped pathway, with pairs of axons initially growing straight down into the musculature, which allows easy phenotypic analysis.^41^ Here, we used fish expressing the HB9:GFP construct to facilitate analysis of primary motor neuron outgrowth occurring in the first 24 hours post-fertilisation (hpf).^41^ Knockdown of endogenous C9orf72 using an ATG-targeting morpholino (C9orf72 MO) and overexpression of mutant TDP-43^G348C^ (mutTDP-43 OE) have both been found to generate similar phenotypes of increased branching and shorter axon lengths in the primary motor neurons at around 30 hpf.^33^^,^^34^

**Figure 1:**
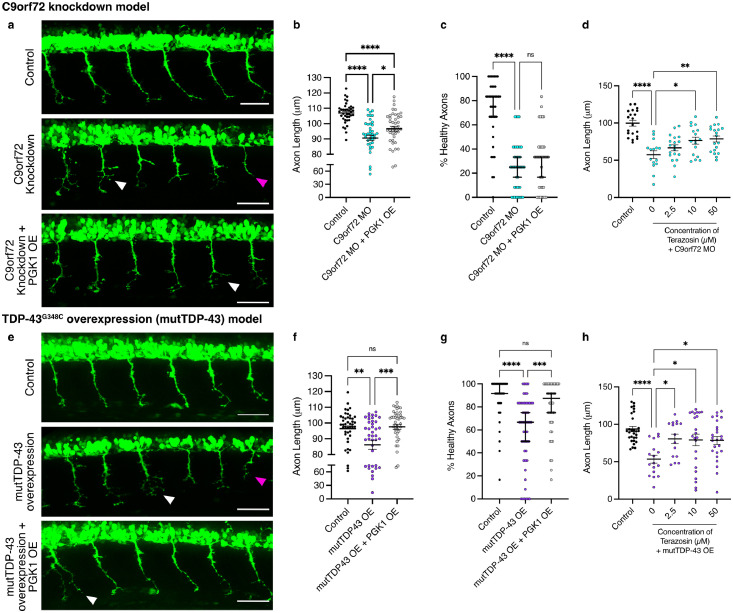
**Targeting PGK1 genetically or pharmacologically improves motor axon phenotypes in zebrafish ALS models.** (a) Representative fluorescent micrographs showing HB9:GFP+ve primary motor neuron outgrowth in uninjected controls, with morpholino-induced C9orf72 knockdown and with co-overexpression of PGK1. Knockdown of C9orf72 produces the motor axon phenotype of shorter axons (magenta arrows) and increased branching (white arrows). Scale bar=50 µM (b) C9orf72-knockdown (C9orf72 MO) decreases mean axon length and overexpression of PGK1 partially rescues this phenotype (one-way ANOVA *P* < 0.0001 with Tukey's multiple comparison test: Control vs C9orf72 MO *P* < 0.0001, C9orf72 MO vs C9orf72 MO + PGK1 OE *P* = 0.016; *n* = 39-45 per treatment group, *n* = 3 clutches). (c) Number of axons scored as “healthy”, i.e. unbranched, in branching analysis. PGK1 overexpression does not change the branching phenotype in C9orf72 MO fish (Kruskal-Wallis *P* < 0.0001 with Dunn's multiple comparison test: Control vs C9orf72 MO *P* < 0.0001, C9orf72 MO vs C9orf72 MO + PGK1 OE *P* = 0.89; *n* = 39-45 per treatment group, *n* = 3 clutches). (d) C9orf72 MO larvae treated with increasing concentrations of terazosin. Terazosin significantly increases axon lengths at 10 µM and 50 µM (one-way ANOVA *P* < 0.0001 with Dunnett's multiple comparison test: Control vs C9orf72 MO *P* < 0.0001, C9orf72 MO vs 2.5 mM terazosin *P* = 0.40, C9orf72 MO vs 10 mM terazosin *P* = 0.02, C9orf72 MO vs 50 mM terazosin *P* = 0.005; *n* = 15-20 per treatment, *n* = 3 clutches). (e) Micrographs showing HB9:GFP+ve primary motor neuron outgrowth in uninjected controls, with mutant TDP-43G348C (mutTDP-43) overexpression and with co-overexpression of PGK1. Overexpression of mutTDP-43 produces the motor axon phenotype of shorter axons (magenta arrows) and increased branching (white arrows). Scale bar=50 µM. (f) mutTDP-43 overexpression (OE) decreases mean axon length and overexpression of PGK1 rescues this phenotype (one-way ANOVA *P* = 0.0003 with Tukey's multiple comparison test: Control vs mutTDP43 OE *P* = 0.003, mutTDP43 OE vs mutTDP43 OE + PGK1 OE *P* = 0.0006, Control vs mutTDP43 OE + PGK1 OE *P* = 0.92; *n* = 44-51 per treatment, *n* = 3 clutches). (g) Number of axons scored as “healthy”, i.e. unbranched, in branching analysis. PGK1 overexpression significantly increases the number of “healthy” axons in the mutTDP-43 OE larvae (Kruskal-Wallis *P* < 0.0001 with Dunn's multiple comparison test: Control vs mutTDP43 OE *P* < 0.0001, mutTDP43 OE vs mutTDP43 OE + PGK1 OE *P* = 0.0006, Control vs mutTDP43 OE + PGK1 OE *P* = 0.13; *n* = 44-51 per treatment, *n* = 3 clutches). (h) mutTDP-43 OE larvae treated with increasing concentrations of terazosin. Terazosin significantly increases axon lengths at 2.5 mM, 10 µM and 50 µM (one-way ANOVA *P* = 0.0002 with Dunnett's multiple comparison test: Control vs mutTDP43 OE *P* < 0.0001, mutTDP43 OE vs 2.5 mM terazosin *P* = 0.017, mutTDP43 OE vs 10 mM terazosin *P* = 0.012, mutTDP43 OE vs 50 mM terazosin *P* = 0.0120; *n* = 15-29 per treatment, *n* = 3 clutches). Error bars represent s.e.m., ns=non-significant, * = *P* < 0.05, ** = *P* < 0.01, *** = *P* < 0.001, **** = *P* < 0.0001.

Overexpression of human PGK1 alone did not have any effect on axon length or branching score (*P* = 0.4575 and *P* = 0.8972 respectively, Supplementary Figure 1a-c). In C9orf72 MO-injected larvae, motor axons at 30 hpf were shorter and more branched than in controls (Figure 1a; magenta and white arrows respectively). Motor axon phenotypes were significantly ameliorated by overexpression of human PGK1, with an increase in axon length (*P* = 0.016; Figure 1b) but no statistically significant change in the percentage of unbranched, healthy axons (*P* = 0.89; Figure 1c) was observed. mutTDP-43 OE produced similar motor axon phenotypes, with decreased axon length and increased branching (Figure 1e), yet overexpression of PGK1 fully rescued this phenotype with an increase in both axon length (*P* = 0.0006; Figure 1f) and the percentage of healthy axons (*P* = 0.0006; Figure 1g). Genetic overexpression of PGK1 was therefore sufficient to ameliorate motor neuron phenotypes in two, genetically distinct, zebrafish models of ALS.

As genetic overexpression of PGK1 does not in itself represent an immediately-translatable treatment option, we next investigated the therapeutic potential of terazosin, an FDA-approved α1-adrenergic receptor antagonist with an established non-canonical action of increasing PGK1 activity.^30^^,^^31^ Increasing concentrations of terazosin were added to the water of developing embryos in each model from 6 hpf until analysis at 30 hpf. Terazosin treatment alone did not have an effect on either branching score or axon length (*P* > 0.999 and *P* = 0.3260 respectively; Supplementary Figure 1d-f). Treatment with terazosin showed a dose-dependent increase in axon lengths in the C9orf72 MO larvae (*P* = 0.005 at 50 µM; Figure 1d) and a significant increase in axon length even at the lowest concentration in mut-TDP-43 OE larvae (*P* = 0.017 at 2.5 µM; Figure 1h). Thus, targeting PGK1, either genetically through overexpression or pharmacologically using terazosin, can rescue key motor neuron phenotypes in ALS zebrafish models.

### Targeting PGK1 improves motor function in ALS models

**Figure 2:**
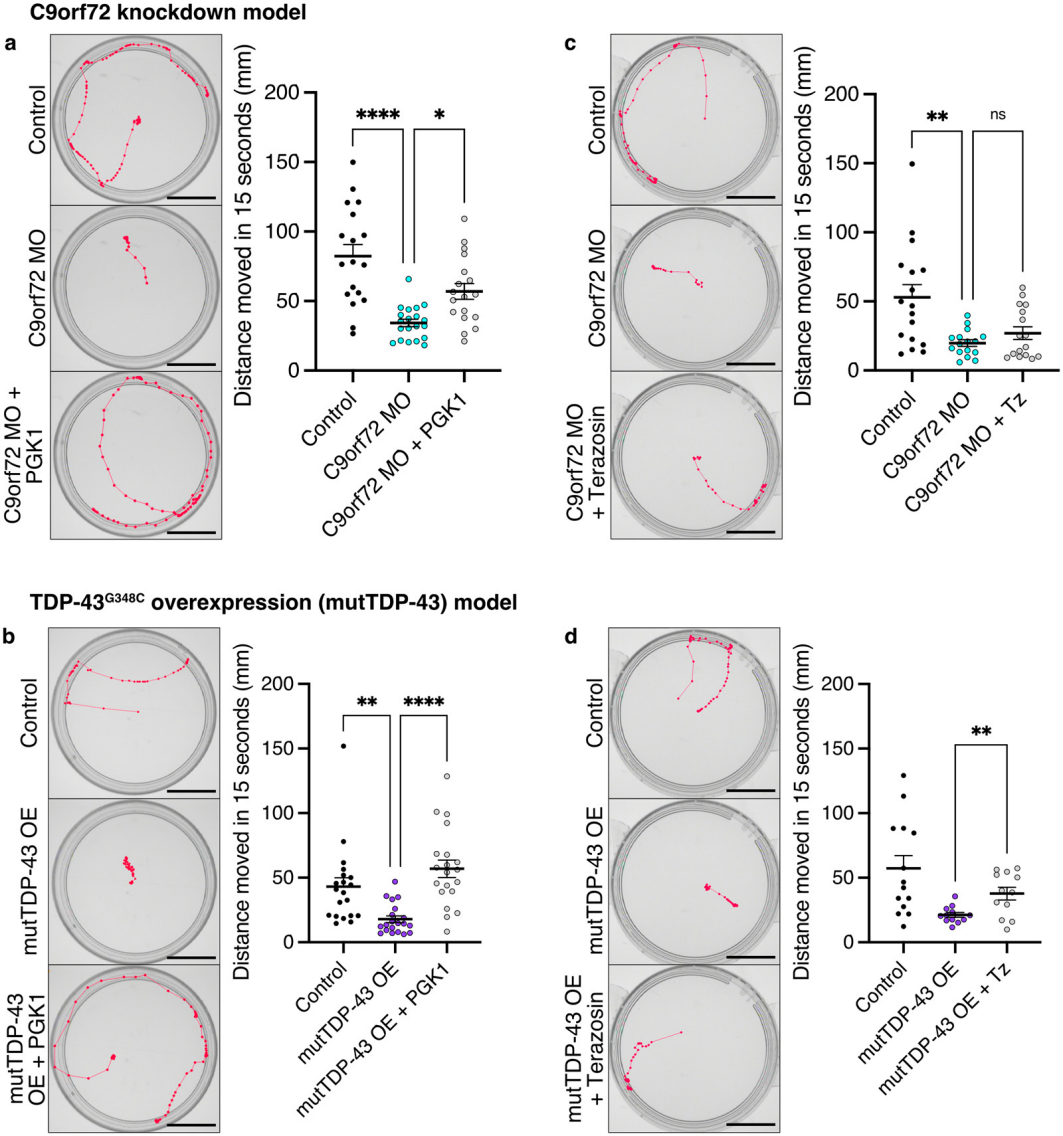
**Targeting PGK1 improves motor function in zebrafish ALS models at 72 hpf.** (a) Results and representative path traces from the TEER (touch-evoked escape response) test in C9orf72 MO larvae following PGK1 overexpression show a significant increase in distance moved following a tail touch (One-way ANOVA *P* < 0.0001 with Tukey's multiple comparison test: Control vs C9orf72 MO *P* < 0.0001, C9orf72 MO vs C9orf72 + PGK1 OE *P* = 0.02). Scale bar=10 mm. (b) Results and representative path traces from the TEER (touch-evoked escape response) test in mutTDP43 OE larvae following PGK1 overexpression show a significant increase in distance moved following a tail touch (One-way ANOVA *P* < 0.0001 with Tukey's multiple comparison test: Control vs mutTDP-43 OE *P* = 0.009, mutTDP-43 OE vs mutTDP-43 OE + PGK1 OE *P* < 0.0001). Scale bar=10 mm. (c) Results and representative path traces from the TEER test in C9orf72 MO larvae following daily terazosin (Tz) shows no difference in distance moved (One-way ANOVA *P* = 0.001 with Tukey's multiple comparison test: Control vs C9orf72 MO *P* = 0.001, C9orf72 MO vs C9orf72 + Tz *P* = 0.70). Scale bar=10 mm. (d) Results and representative path traces from the TEER test in mutTDP-43 OE larvae following daily terazosin (Tz) treatment shows a significant increase in distance moved following a tail touch (unpaired t-test, *P* = 0.0047). Scale bar=10 mm. Each point represents the average of 3 trials from a single larva. *n* = 2 clutches, *n* = 12-20 per group. Error bars represent s.e.m., ns=non-significant, * = *P* < 0.05, ** = *P* < 0.01, **** = *P* < 0.0001.

At 72 hpf, zebrafish larvae show a robust tail-touch evoked escape response (TEER) which can be quantified by the distance moved following the tail touch, and is dramatically reduced in ALS models.^34^ Therefore, we utilised this experimental paradigm to establish whether changes in motor neuron growth resulting from targeting PGK1 were sufficient to generate concomitant improvements in motor function in vivo (Supplementary Movies 1 & 2). Both larval ALS zebrafish models were treated as detailed above, with either PGK1 overexpression at the single cell stage or treatment with 50 mM terazosin from 6 hpf, and allowed to develop until 72 hpf (n = 12-20 larvae across 2 clutches per treatment group). As expected, induction of either ALS model led to a significant decrease in the total distance travelled in the TEER test (*P* < 0.0001 for C9orf72 MO model, *P* = 0.009 for mutTDP-43 OE model; Figure 2a & b). These motor phenotypes were rescued following PGK1 overexpression, where there was a significant increase in distance moved in both C9orf72 MO larvae (*P* = 0.02; Figure 2a) and mutTDP-43 OE larvae (*P* < 0.0001; Figure 2b). Although there was no significant change in TEER distances in the C9orf72 MO following daily treatment of terazosin (*P* = 0.70; Figure 2c), there was a significant increase in distance moved by mutTDP-43 OE larvae following treatment with 50 µM terazosin (*P* = 0.0047; Figure 2d). We therefore show that genetic overexpression of PGK1 can improve motor function in two zebrafish models of ALS, while treatment with terazosin significantly improves motor function in the mutTDP43 OE model.

### Terazosin treatment increases survival and improves clinical phenotypes in a TDP-43 mouse model by protecting against motor neuron death

In order to establish whether the therapeutic effects of targeting PGK1 in zebrafish models of ALS could be extended into the mammalian neuromuscular system, we next turned to a TDP-43 mouse model of ALS. The hTDP-43 mouse model expresses the human *TARDBP* gene under the *Thy1* promoter. This neuron-specific expression of TDP-43 leads to a very fast-progressing ALS model, with homozygous hTDP-43 mice showing a clinical phenotype of progressive hindlimb paralysis, body weight loss and a life expectancy of ∼20-25 days with TDP-43 pathology and motor neuron cell death in the spinal cord.^42^ hTDP-43 mice were treated daily with either 10 or 100 µg/kg terazosin, or saline control, and assessed for changes in clinical score and body weight. A clinical score of 1 indicates a mild paralysis phenotype, with weakness in one hindlimb. A clinical score of 2 indicates a moderate paralysis phenotype with evident paralysis in both hindlimbs, whilst a clinical score of 3 indicates complete paralysis in both hindlimbs (representing the humane endpoint for this model).

**Figure 3:**
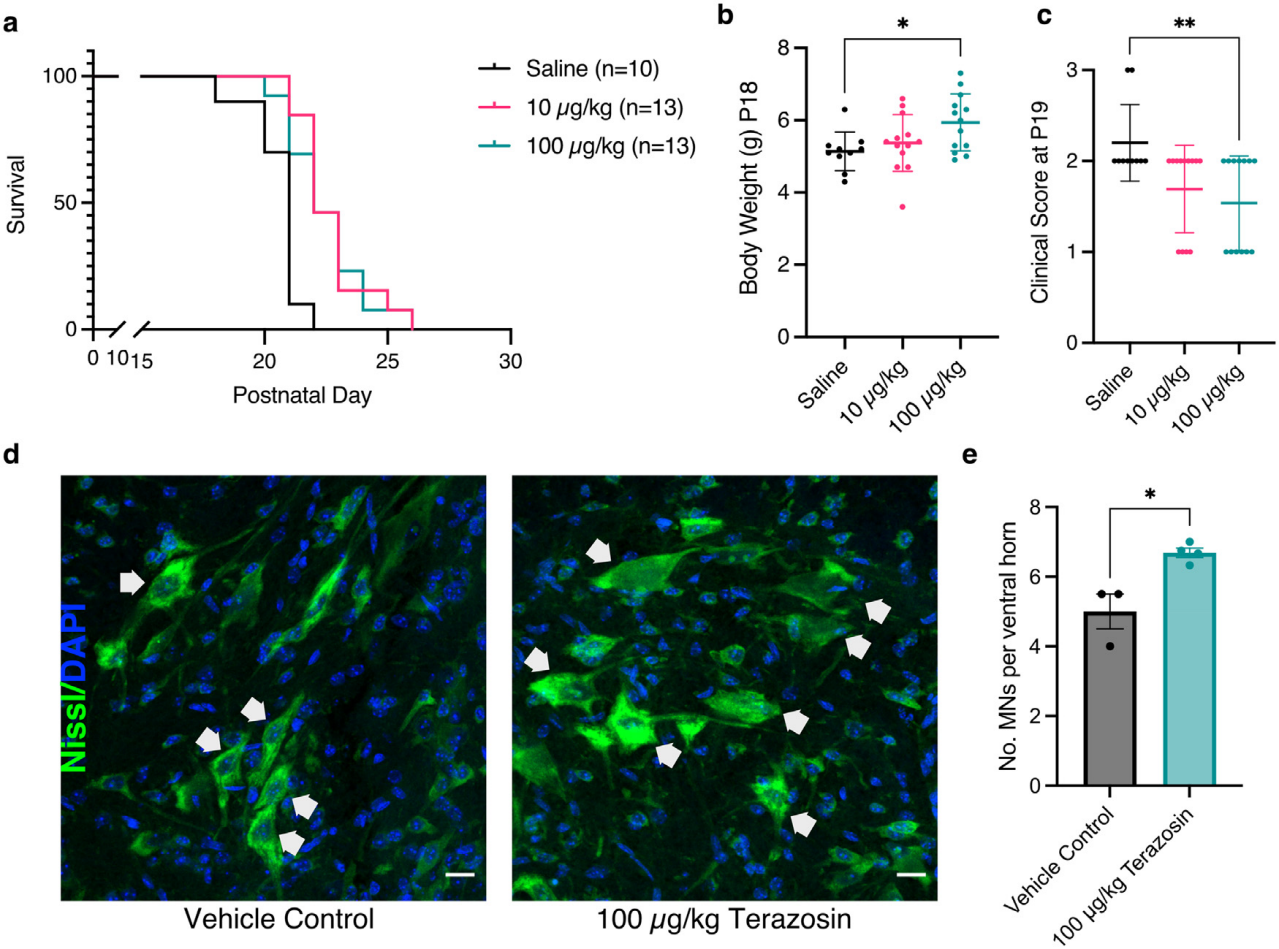
**Treating hTDP-43 mice with terazosin significantly improves survival and clinical phenotypes.** (a) Survival analysis for hTDP-43 mice shows a significant increase in survival when treated with 10 µg/kg (*P* = 0.0002; Log-rank Mantel-Cox test) or 100 µg/kg terazosin (*P* = 0.0023; Log-rank Mantel-Cox test) compared to vehicle controls. (b) Mice treated with 100 µg/kg terazosin show a significant increase in body weight compared to saline controls (n = 10-13 per group; One-way ANOVA *P* = 0.033 with Dunnett's multiple comparison test: Saline vs 10 µg/kg *P* = 0.67, Saline vs 100 µg/kg *P* = 0.025). (c) Clinical scores per treatment group at P19. Mice treated with 100 µg/kg terazosin show a significantly lower clinical score at P19 compared to saline controls (*n* = 10-13 per group; Kruskal-Wallis test *P* = 0.014 with Dunn's multiple comparison test: Saline vs 10 µg/kg *P* = 0.065, Saline vs 100 µg/kg *P* = 0.009). (d) Representative micrographs of lumbar spinal cord sections from hTDP-43 mice treated with saline vehicle control or 100 µg/kg terazosin. Motor neurons were defined as cells in the ventral horn with the shortest diameter >20 µm, represented by white arrows. Scale bar=20 µm. (e) Quantification of number of motor neurons shows a significant increase in motor neuron number following terazosin treatment (t-test *P* = 0.0132; *n* = 3-4 mice per treatment group, each point = average of 4-6 ventral horns) Error bars represent s.e.m., * = *P* < 0.05, ** = *P* < 0.01. Each point represents one mouse.

Treatment with terazosin moderately but significantly improved survival by 5% at both mid and high doses from a median survival of 21 to 22 days in both treatment groups (*P* = 0.0002 for 10 µg/kg terazosin and *P* = 0.0023 for 100 µg/kg; Figure 3a). In wild-type control littermates, mice did not develop any hindlimb paralysis or reach a humane endpoint over the course of the experiment, and terazosin had no effect on either survival or body weight (Supplementary Figure 2). At P18, when all mice were displaying disease phenotypes, there was a significant increase of 0.8 g in body weight in mice treated with 100 µg/kg terazosin compared to saline controls (*P* = 0.025; Figure 3b). Importantly, these mice also showed a significant decrease in mean clinical score (*P* = 0.009; Figure 3c). The progressive paralysis in hTDP-43 mice corresponds with motor neuron cell death in the spinal cord.^42^ Mice treated with 100 µg/kg terazosin versus saline control were assessed at the symptomatic time point of P19 for motor neuron number in the lumbar spinal cord. Spinal cord sections were stained for Nissl using a fluorescent dye, and neurons in the ventral horn with the shortest diameter of >20 µm were counted as alpha motor neurons. Treatment with terazosin seems to protect against motor neuron cell death, with a 40% increase in number of motor neurons per ventral horn following terazosin treatment compared to vehicle treated control (*P* = 0.0132; Figure 3d & e). Terazosin treatment had no effect on motor neuron number in the ventral horn of wild type mice (*P* = 0.7428; Supplementary Figure 2). Thus, even in a very severe mouse model of TDP-43 overexpression, treatment with terazosin is neuroprotective with significant improvements in both survival and clinical phenotypes resulting from protection of motor neuron death.

### Terazosin improves cell survival in TDP-43^M337V^ ESC-MNs

**Figure 4:**
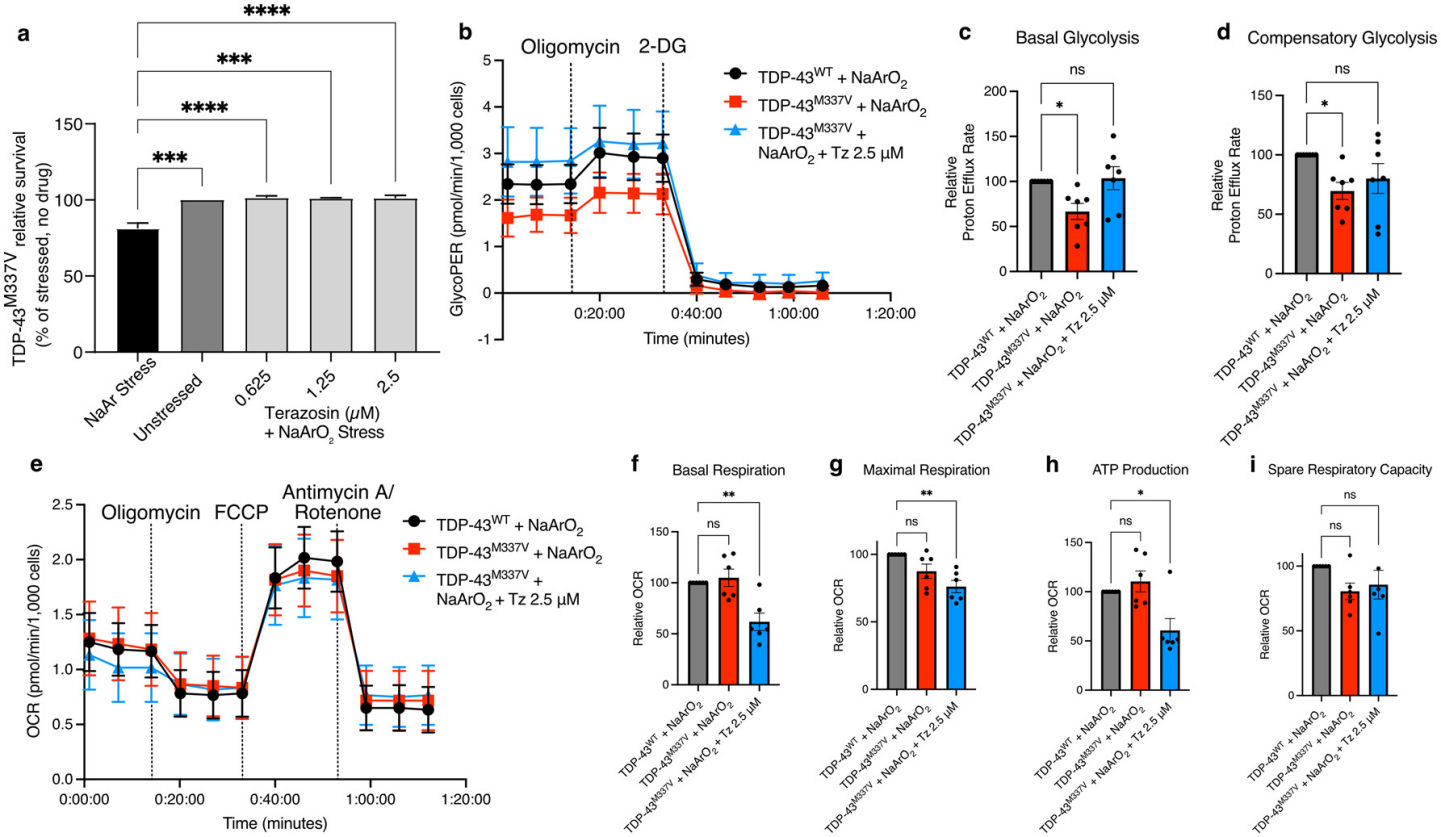
**Terazosin protects against NaArO_2_-induced cell death, increases glycolysis and decreases mitochondrial respiration in mESC-MNs**. (a) TDP-43^M337V^ mESC-MNs show a decreased survival in response to sodium arsenite (NaArO_2_) stress compared to unstressed controls. Survival following terazosin treatment at all concentrations is maintained at 100%. (*n* = 3 differentiations; One-way ANOVA *P* < 0.001 with Dunnett's multiple comparisons). (b) Traces from the Seahorse analyser glycolytic rate assay showing glycolytic proton efflux rate (GlycoPER) following mitochondrial inhibition (oligomycin) and inhibition of the glycolysis pathway (2-deoxy-D-glucose; 2-DG). (c) TDP-43^M337V^ mESC-MNs demonstrate a significantly lower rate of basal glycolysis compared to TDP-43^WT^ controls, which is rescued by treatment of 2.5 µM terazosin (Tz) (One-way ANOVA *P* = 0.017 with Bonferroni's multiple comparison test: TDP-43^WT^ + NaArO_2_ vs TDP-43^M337V^ + NaArO_2_*P* = 0.034, TDP-43^WT^ + NaArO_2_ vs TDP-43^M337V^ + NaArO_2_ + 2.5 µM Tz *P* > 0.99). (d) TDP-43^M337V^ mESC-MNs show significantly reduced rates of compensatory glycolysis compared to TDP-43^WT^ controls, which is rescued by treatment of 2.5 µM terazosin (Tz) (One-way ANOVA *P* = 0.055 with Bonferroni's multiple comparison test: TDP-43^WT^ + NaArO_2_ vs TDP-43^M337V^ + NaArO_2_*P* = 0.038, TDP-43^WT^ + NaArO_2_ vs TDP-43^M337V^ + NaArO_2_ + 2.5 µM Tz *P* = 0.22). (e) Traces from the Seahorse analyser showing oxygen consumption rate (OCR) following mitochondrial inhibition (oligomycin), mitochondrial uncoupling (FCCP) and electron transport chain inhibition (antimycin A/rotenone). (f) Treatment with 2.5 µM terazosin significantly decreases basal respiration in TDP-43^M337V^ mESC-MNs compared to TDP-43^WT^ controls (One-way ANOVA *P* = 0.001 with Bonferroni's multiple comparison test: TDP-43^WT^ + NaArO_2_ vs TDP-43^M337V^ + NaArO_2_*P* > 0.99, TDP-43^WT^ + NaArO_2_ vs TDP-43^M337V^ + NaArO_2_ + 2.5 µM Tz *P* = 0.003). (g) Treatment with 2.5 µM terazosin significantly decreases maximal respiration in TDP-43^M337V^ mESC-MNs compared to TDP-43^WT^ controls (One-way ANOVA *P* = 0.003 with Bonferroni's multiple comparison test: TDP-43^WT^ + NaArO_2_ vs TDP-43^M337V^ + NaArO_2_*P* = 0.091, TDP-43^WT^ + NaArO_2_ vs TDP-43^M337V^ + NaArO_2_ + 2.5 µM Tz *P* = 0.002). (h) Treatment with 2.5 µM terazosin significantly decreases ATP production in TDP-43^M337V^ mESC-MNs compared to TDP-43^WT^ controls (One-way ANOVA *P* = 0.004 with Bonferroni's multiple comparison test: TDP-43^WT^ + NaArO_2_ vs TDP-43^M337V^ + NaArO_2_*P* = 0.87, TDP-43^WT^ + NaArO_2_ vs TDP-43^M337V^ + NaArO_2_ + 2.5 µM Tz *P* = 0.018). (i) Treatment with 2.5 µM terazosin does not change the spare respiratory capacity in TDP-43^M337V^ mESC-MNs compared to TDP-43^WT^ controls (One-way ANOVA *P* = 0.19 with Bonferroni's multiple comparison test: TDP-43^WT^ + NaArO_2_ vs TDP-43^M337V^ + NaArO_2_*P* = 0.16, TDP-43^WT^ + NaArO_2_ vs TDP-43^M337V^ + NaArO_2_ + 2.5 µM Tz *P* = 0.38). Each data point represents a separate differentiation; *n* = 7 per glycolysis analysis; *n* = 6 per respiration analysis. Error bars represent s.e.m., ns=non-significant, * = *P* < 0.05, ** = *P* < 0.01.

We hypothesised that the improvements in motor function, survival and motor neuron phenotypes seen in animal models of ALS following terazosin treatment were due to neuroprotective mechanisms. To further investigate this, we assessed the impact of terazosin treatment in mouse embryonic stem-cell derived motor neurons (mESC-MNs) expressing mutant TDP-43^M337V^. These mESC-MNs express human *TARDBP* with the M337V mutation under the human promoter and produce low levels of the human TDP-43 protein.^37^ Analysis of mESC-MNs allows high-throughput *in vitro* analysis and the low TDP-43 expression in this model allows us to study toxic gain-of-function associated with the mutation, rather than overexpression of the protein. TDP-43^M337V^ mESC-MNs show cytoplasmic mislocalisation of TDP-43 protein and are particularly susceptible to sodium arsenite (NaArO_2_)-induced oxidative stress, which induces cell death (Figure 4a). TDP-43^M337V^ ESC-MNs were incubated with increasing doses of terazosin for 24 hours prior to NaArO_2_ stress. Terazosin treatment was found to be neuroprotective, with a complete rescue of survival at 100% following stress activation (*P* < 0.001; Figure 4a). Thus, it is likely that terazosin confers motor neuron protection in ALS, at least in part, by modulating oxidative stress responses. This is consistent with a role for PGK1 in regulating oxidative stress pathways.^43^

### Terazosin increases basal glycolysis and reduces mitochondrial respiration in motor neurons

Since terazosin is known to increase PGK1 activity,^30^^,^^31^^,^^44^ we hypothesised that the therapeutic effects observed in ALS models may be occurring due to changes in glycolysis and/or respiration. We therefore performed Seahorse analyses to assess glycolysis and respiration rates following oxidative stress in TDP-43^M337V^ mESC-MNs compared to TDP-43^WT^ controls (Figure 4b & e). mESC-MNs from the TDP-43^WT^ mouse line have been reported to have no pathology in terms of TDP-43 mislocalisation or altered stress granule dynamics^37^ and so make a useful control for TDP-43^M337V^ mESC-MNs in terms of transgene expression. Using this technique, we assessed the rate of basal glycolysis and of compensatory glycolysis following mitochondrial inhibition. To compare the effects of terazosin treatment on glycolysis and respiration output, each differentiation was normalised to TDP-43^WT^ mESC-MN control. TDP-43^M337V^ mESC-MNs show a reduction in both basal glycolysis (*P* = 0.034; Figure 4c) and compensatory glycolysis compared to TDP-43^WT^ controls (*P* = 0.038; Figure 4d). Following 24 hours of incubation with 2.5 µM terazosin, there was a significant increase in both basal glycolysis and compensatory glycolysis to levels comparable with those observed in TDP-43^WT^ mESC-MNs (*P* > 0.99 and *P* = 0.22 respectively; Figure 4c and d). We next used the Seahorse Mito Stress Test to assess basal mitochondrial respiration, maximal respiration, ATP production and spare respiratory capacity (Figure 4 e-i). TDP-43^M337V^ mESC-MNs did not demonstrate any significant differences in mitochondrial respiration parameters compared to TDP-43^WT^ mESC-MN controls. However, when treated with 2.5 µM terazosin, TDP-43^M337V^ mESC-MNs showed significantly lower basal and maximal respiration rates (*P* = 0.003 and *P* = 0.002 respectively; Figure 4f & g) as well as lower ATP production from mitochondrial respiration (*P* = 0.018; Figure 4h). This increase in glycolysis and decrease in mitochondrial respiration indicates a metabolic switch, which may be part of the mechanism through which terazosin exerts its neuroprotective action. This appears to be dependent on the oxidative stress environment, since unstressed cells treated with terazosin did not show any significant differences in glycolysis or mitochondrial respiration compared to controls (Supplementary Figure 3).

### Terazosin restores stress granule formation in ESC-MNs

**Figure 5:**
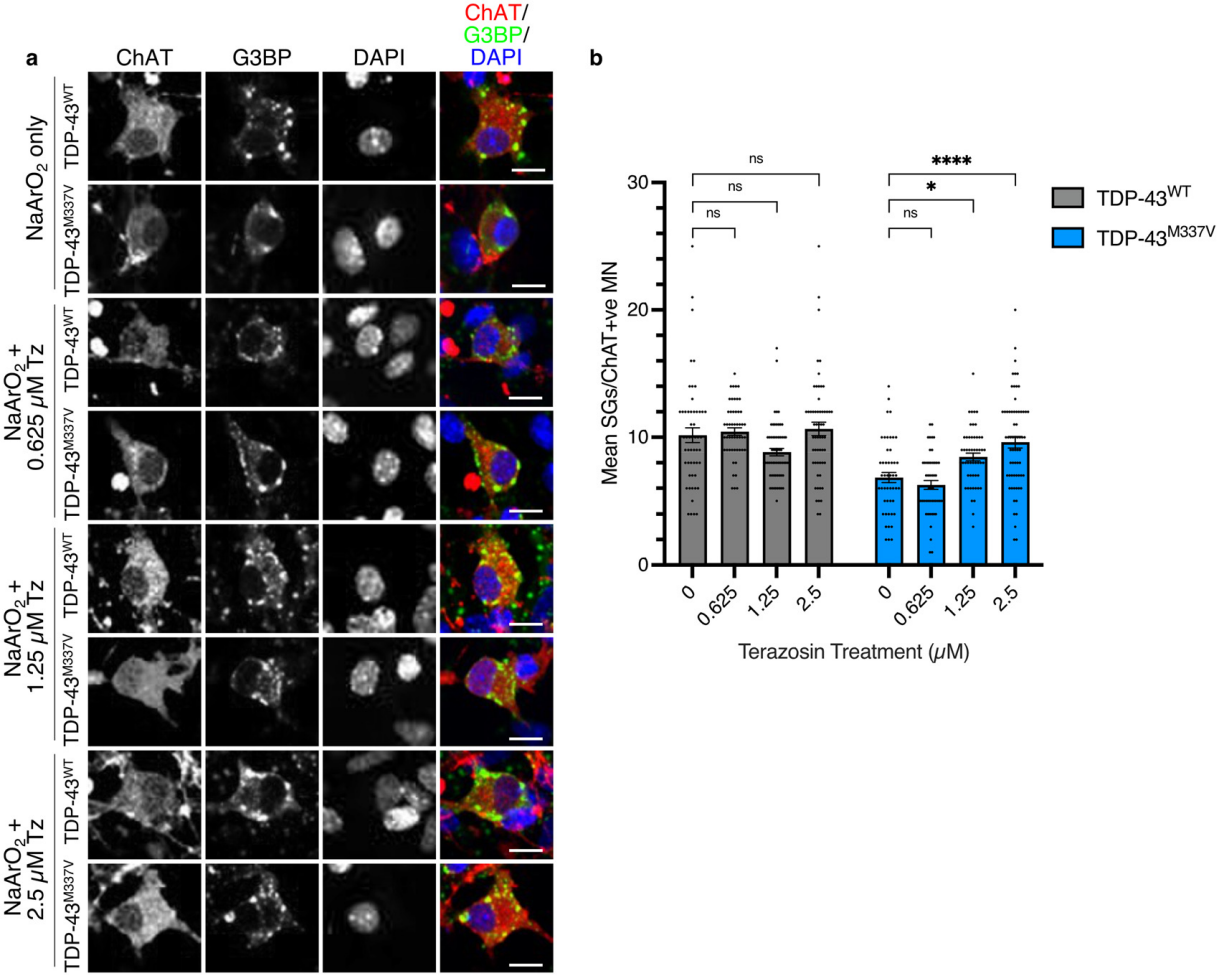
**Terazosin rescues stress granule formation.** (a) Images of TDP-43^WT^ and TDP-43^M337V^ mESC-MNs following NaArO_2_-induced oxidative stress with treatment of increasing doses of terazosin. Red channel shows ChAT (motor neuron marker), green channel shows G3BP (stress granule marker), blue channel shows DAPI (nuclear marker). (b) TDP-43^M337V^ mESC-MNs show an impairment in the ability to produce stress granules compared to TDP-43^WT^ controls. Treatment with terazosin has no effect on stress granule formation in TDP-43^WT^ mESC-MNs (One-way ANOVA *P* = 0.013 with Dunnett's multiple comparisons: 0 µM Tz vs 0.625 µM Tz *P* = 0.95; 0 µM Tz vs 0.125 µM Tz *P* = 0.081; 0 µM Tz vs 2.5 µM *P* = 0.77), but increases the mean number of stress granules per motor neuron in TDP-43^M337V^ mESC-MNs in a dose-dependent manner (One-way ANOVA *P* < 0.0001 with Dunnett's multiple comparisons: 0 µM Tz vs 0.625 µM Tz *P* = 0.63; 0 µM Tz vs 0.125 µM Tz *P* = 0.015; 0 µM Tz vs 2.5 µM *P* < 0.0001). N = 3 differentiations, n > 30 random cells per differentiation. Error bars represent s.e.m., ns=non-significant, * = *P* < 0.05, *** = *P* < 0.001.

As an RNA binding protein, TDP-43 is involved in a number of important regulatory RNA processes, including stress granule formation which occurs in response to stressed cellular conditions.^45^ Since TDP-43 pathology is seen in 97% of ALS cases,^46^ therapies that target these pathways could be relevant for patients with multiple forms of ALS. We previously reported that formation of stress granules is impaired in TDP-43^M337V^ mESC-MNs.^37^ Since terazosin treatment has a neuroprotective effect on motor neurons following oxidative stress-induced injury (Figure 4a), we assessed whether terazosin had an effect on this critical subcellular phenotype. mESC-MNs expressing either TDP-43^WT^ or TDP-43^M337V^ were incubated with increasing doses of terazosin for 24 hours before being fixed and probed for motor neuron and stress granule markers. The number of stress granules per motor neuron were then counted (Figure 5a). Treatment with terazosin showed no effect on stress granule assembly in TDP-43^WT^ mESC-MNs (Figure 5b). However, in TDP-43^M337V^ mESC-MNs, which show an impairment in stress granule formation, there was a dose-dependent increase in the number of stress granules per motor neuron at 1.25 µM and 2.5 µM terazosin, and full rescue of stress granule formation at the highest dose when compared to TDP-43^WT^ mESC-MNs (*P* =  0.015 and *P* < 0.0001 respectively; Figure 5b). This suggests an additional mechanism of action for terazosin in recovery of stress granule formation, a molecular pathology common across multiple ALS models and therefore more likely to be of benefit to multiple ALS patient groups regardless of genetic background.

## Discussion

Targeting bioenergetic pathways represents an attractive opportunity for the treatment of neurodegenerative disease. Here, we demonstrate that directly targeting PGK1 activity, including via treatment with terazosin, has the potential to act as a neuroprotective agent in ALS, ameliorating disease phenotypes both *in vivo* and *in vitro* across species and gene mutations. Both overexpression of PGK1 and treatment with terazosin improved key motor neuron phenotypes in zebrafish with knockdown of C9orf72 or overexpression of mutant TDP-43. Importantly, this then correlated with significant improvements in motor performance and behaviour. The therapeutic effects of terazosin translated to mammalian models of TDP-43 overexpression, where treatment led to an increase in median survival in the severe Thy1-hTDP-43 mouse model, with corresponding increases in body weight and improvement in clinical scores. At a cellular level, terazosin was neuroprotective of the motor neuron death seen in this mouse model, with an increase in motor neurons in the lumbar spinal cord at a symptomatic time point. We further showed that terazosin is neuroprotective in TDP-43^M337V^ mESC-MNs, where treatment rescued the cell death seen following sodium arsenite exposure. Terazosin treatment increased rates of glycolysis and decreased rates of mitochondrial respiration indicating that terazosin is acting through changes in bioenergetic pathways, while also restoring stress granule formation.

Since the first point mutations to be associated with ALS were found in the mitochondrial-linked enzyme *SOD1*,^47^ mitochondrial dysfunction has long been a focus of ALS research. Indeed, an increased number of mitochondrial DNA mutations and a lower amount of mitochondrial DNA, directly related to mitochondrial number, were found in spinal cords of sporadic ALS patients compared to controls.^48^ Subsequently, mitochondrial dysfunction has been seen in models of *SOD1, C9orf72, TDP-43* and *FUS* mutations,^49^, ^50^, ^51^, ^52^, ^53^ as well as post-mortem patient samples.^18^ However, targeting energy metabolism has yet to deliver on its full therapeutic potential for ALS. Indeed, drugs targeting either mitochondria or reactive oxygen species production have seemed promising in animal models but have gone on to fail in subsequent clinical trials. For example, olesoxime was shown to delay several disease phenotypes in the SOD1^G93A^ ALS mouse model,^54^ yet failed to change median survival or ALS-FRS-R score in a Phase II-III study.^55^ One facet of this failure to translate to the clinic is the reliance on the SOD1^G93A^ mouse, a model that represents only a small fraction of ALS patients. Researchers have since learnt that the heterogeneous nature of ALS, both in terms of clinical presentation and genetic background, necessitates that novel compounds should ideally be tested across multiple ALS models for better translation to the clinic. A recent consensus committee highlighted the importance of focusing on models that recapitulate phenotypes seen in sporadic ALS in order to provide a strong biological rationale for novel therapies as well as evidence of target engagement.^56^ This is a strategy that we have used in this study, where we have described the neuroprotective effect of terazosin across multiple phenotypes in models based on both C9orf72 and TDP-43, while providing two potential mechanisms of action.

While we have shown that terazosin is broadly neuroprotective across ALS models, it has also been tested in various other neurodegenerative models. In *Drosophila* models of hypoxia, terazosin increased survival, while IP injection of terazosin in rodent models prior to middle cerebral artery occlusion reduced infarct volume size.^30^ Terazosin has recently been shown to ameliorate disease phenotypes across multiple mouse models of Parkinson's disease, as well as in *Drosophila* and patient iPSC-MNs.^31^ Interestingly, it has recently been shown that intracerebroventricular injection of Pgk1 is protective against MPTP-induced toxicity in zebrafish, where Pgk1 treatment rescued both cellular and behavioural phenotypes.^57^ Indeed, extracellular Pgk1, possibly released from muscle, may have a role in neurite outgrowth via the cofilin pathway.^58^ We have previously shown that *Pgk1* expression is differentially regulated between motor neurons innervating vulnerable muscles versus those innervating resistant muscles in the childhood-onset motor neuron disease spinal muscular atrophy (SMA).^29^ We showed that both *PGK1* overexpression and terazosin treatment was therapeutic in zebrafish models of SMA, with reduced branching phenotypes.^29^ Terazosin has even been shown to be therapeutic in non-neurodegenerative indications such as gastrointestinal disorders.^43^ The work presented here therefore builds on this body of evidence showing that terazosin can protect against degeneration across multiple diseases, and may be particularly important in the context of hard-to-treat neurodegenerative disorders due to the fact that terazosin can cross the blood-brain barrier.^31^

Terazosin is an attractive candidate for therapy development due to its known safety profile in adults. Interestingly, studies have found a biphasic dose response of terazosin, where doses in rodent models above around 100 µg/kg lose their neuroprotective effect.^30^^,^^31^ We therefore restricted the doses in mouse models of ALS to a maximum of 100 µg/kg (Figure 3). Although we did not see a biphasic dose response in analysis of axon length in our zebrafish models (Figure 1), this may be due to the barrier of the chorion in the zebrafish eggs, since while we added a known concentration of terazosin to the fish water, it is unknown how much terazosin in fact reached the zebrafish embryo. Equally, due to the change in methods of administration between zebrafish and rodent models, it is possible that we simply did not reach a high enough concentration in our zebrafish work to see this curve. Regardless, this loss of neuroprotection at higher doses will need to be considered if this moves towards the clinic. Current evidence that terazosin is neuroprotective in patients comes from retrospective analysis of databases of Parkinson's disease patients. Due to low numbers of patients being prescribed terazosin, mainly for benign prostatic hyperplasia, the “treated” groups have included those patients prescribed doxazosin or alfuzosin since they also contain the predicted PGK1-binding domain, whereas tamsulosin is used as an α1-adrenergic receptor antagonist structurally distinct from terazosin without a known PGK1 binding domain. Initial assessment of 13 patients treated with terazosin, doxazosin or alfuzosin in the Parkinson's Progression Markers Initiative database showed a slower rate of motor function decline compared to tamsulosin treated or control.^31^ Expanding to the larger IBM Watson/Truven Health Analytics MarketScan Database, a group of 2,880 Parkinson's patients treated with terazosin/doxazosin/alfuzosin were found to have a reduced relative risk of a Parkinson's diagnosis compared to tamsulosin treated or age-matched controls.^31^ Further work combining the Truven database with national databases in Denmark also found that those patients prescribed terazosin, doxazosin or alfuzosin had a lower hazard of developing Parkinson's disease.^32^ This group has followed up their retrospective analysis with a pilot study treating Parkinson's patients with 5 mg terazosin, where they found elevated levels of ATP in the brain and blood samples following treatment.^44^ These clinical data demonstrate the potential of terazosin as a therapy, particularly since Parkinson's disease is another neurodegenerative disorder with genetic heterogeneity and an unknown initial cause of onset, as in ALS.

While we have provided further evidence to support the broadly protective effects of terazosin via boosting basal glycolysis, we have also shown a potentially interesting additional ALS-specific mechanism in the recovery of stress granule formation (Figure 5). Stress granules are transient assemblies of RNA molecules with RNA binding proteins and are an important element of a healthy cellular response to stress, particularly by inhibiting translation.^59^ Several ALS-associated gene mutations occur in RNA binding proteins, including *FUS, TIA1, HNRNPA2B1* and *TARDBP*, the gene that encodes TDP-43,^6^ which have all been associated with stress granules.^45^^,^^60^, ^61^, ^62^ Conversely, TDP-43-positive inclusions in ALS patient tissue are also positive for stress granule markers^63^ and one hypothesis of the origin of these pathological aggregates is sustained stress granule formation with no disaggregation.^64^ Knockdown of TDP-43 in cell culture reduces formation of stress granules in response to oxidative stress^45^ and some mutations in TDP-43 lead to reduced stress granule formation.^37^^,^^65^ Here we have shown that TDP-43^M337V^ mESC-MNs have fewer stress granules than those from the TDP-43^WT^ model, and that treatment with terazosin rescues this phenotype in a dose-dependent manner (Figure 5). Stress granule assembly and dynamics are ATP dependent.^66^ Since we have shown that terazosin increases the rate of glycolysis, it is possible that while the loss of stress granule assembly is not due to changes in ATP availability, the rescue of stress granule formation following terazosin treatment may be due to an increase in glycolysis.

There are some limitations to our study. Firstly, the models of ALS used here are based on genetic mutations in ALS-associated genes or overexpression of the TDP-43 protein, and have therefore not addressed the sporadic disease context. However, since terazosin is not targeting the genetic onset of disease, but rather providing a neuroprotective environment for motor neurons to overcome key disease phenotypes, we feel that the evidence of a therapeutic effect across multiple ALS models implies that terazosin may also succeed in translating to sporadic ALS. A further limitation to this study is that each ALS model used here uses relatively immature neurons either in the larval stage of zebrafish, in the severe Thy1-hTDP-43 mouse model or in ESC-MNs. The most mature of these models, the Thy1-hTDP-43 mouse model, describes a much more acute disease progression than is seen in ALS. This mouse model may therefore be considered more a model of TDP-43 overexpression. While we have focussed here on the breadth of ALS models, future studies would benefit from longer treatment regimes in models with a later onset of motor phenotypes or alternative genetic mutations. Despite these limitations, we present evidence here that terazosin is an ideal candidate to move forwards as a therapy for ALS. Terazosin has a long history of being prescribed for hypotension and benign prostatic hyperplasia, and so the side effects are known. While there is some evidence of terazosin being cytotoxic to cancer cells,^67^ we did not see any evidence of cytotoxicity in our study and in fact terazosin treatment was protective against oxidative stress in TDP-43^M337V^ mESC-MNs. Some side effects seen in patients include mild dizziness and transiently elevated levels of serum aminotransferases which were not long-term.^68^ While these mild side effects should be considered by clinicians if prescribing terazosin long-term, we believe that there are no barriers in terms of its safety profile before terazosin can be tested in ALS patients.

In conclusion, we have demonstrated the therapeutic potential of terazosin across multiple disease models of ALS. We have shown that terazosin acts in a general neuroprotective manner via an increase in glycolysis, while also acting through ALS-specific molecular mechanism of increasing stress granule formation in response to stress. We therefore propose terazosin as a new therapy to be repurposed for ALS, with the possibility of helping a wide range of motor neuron disease patients if translated to the clinic.

## Contributors

This project was administered by H.C., K.T. and T.H.G. H.C., D.G., T.B., C.B., K.M.E.F., K.T. and T.H.G. conceived, planned and supervised these experiments. H.C., E.C., D.G., Y.T.H., D.vdH., H.L.S., and K.M.E.F. performed laboratory experiments. H.C., E.C. and D.G. performed data analysis. K.M.E.F., K.T. and T.H.G. acquired funding for this study. H.C., K.M.E.F. and T.H.G. wrote the original draft. For zebrafish and mouse work, H.C. and T.H.G. have verified all data. For mESC-MN work, E.C., D.G. and K.T. have verified all data. All authors contributed to reviewing the paper and all authors have read and approved the final version for submission.

## Data sharing statement

Summarised data are available in the main text or the supplementary materials. There are no restrictions on material or data. Raw data are available through request to the corresponding author. For the purpose of open access, the author has applied a creative commons attribution (CC BY) licence to any author accepted manuscript version arising.

## Declaration of interests

H.C. received travel funding from MND Scotland and the My Name’5 Doddie Foundation, and E.C. from Medical Research Council (BRT00030) and Medical Sciences Division Doctoral Training Centre (MSDTC) Student Fellowship (BST0010Z), to attend conferences and communicate this research. The other authors have declared that no conflict of interest exists.

## Appendix Supplementary materials

Image, application 1

Image, application 2

Image, video 3

Image, video 4

Image

Image

Image
