# Supplementary material for: Targeting phosphoglycerate kinase 1 with terazosin improves motor neuron phenotypes in multiple models of amyotrophic lateral sclerosis
Source: eBioMedicine. 2022 Aug 11;83:104202. doi: 10.1016/j.ebiom.2022.104202 (PMC9482929; doi:10.1016/j.ebiom.2022.104202)
Supplement: Supplementary file 1 [file mmc1.docx]

**Supplementary Figure 1. Overexpression of PGK1 or treatment with terazosin does not affect axon length or axonal branching in healthy controls.** (**a**) Micrographs of zebrafish as uninjected controls or injected with a PGK1 overexpression construct (PGK1 OE). Scale bars=50 µM. (**b**) Axon lengths of zebrafish embryos injected with PGK1 overexpression construct (PGK1 OE) compared to uninjected control (unpaired t-test; P=0.4575; n=10). (**c**) Percentage of healthy, unbranched axons in zebrafish embryos injected with PGK1 overexpression construct (PGK1 OE) compared to uninjected control (Mann-Whitney test; P=0.8972; n=10). (**d**) Micrographs of zebrafish as uninjected controls or treated with 25 µM terazosin. Scale bars = 50 µM. (**e**) Axon lengths of zebrafish embryos treated with 25 µM terazosin compared to uninjected control (unpaired t-test; P=0.3260; n=4). (**f**) Percentage of healthy, unbranched axons in zebrafish embryos treated with 25 µM terazosin compared to uninjected control (Mann-Whitney test; P>0.9999; n=4). N.s.=non-significant. Error bars represent S.E.M. Each point represents an average of 12 axons from one embryo.

**Supplementary Figure 2. Terazosin treatment does not affect survival or body weight in wild-type littermate control mice.** (**a**) There were no deaths in wild-type littermates treated with terazosin or saline control through the course of the study. (**b**) There was no change in body weight in wild-type littermates treated with terazosin compared to saline control (one-way ANOVA P=0.7657; Tukey’s post-hoc correction Saline vs 10 µg/kg P=0.6117, Saline vs 100 µg/kg P=0.6621, 10 µg/kg vs 100 µg/kg P=0.2466; n=6-11). (**c**) Representative micrographs of lumbar spinal cord sections from wild-type littermate control mice treated with saline vehicle control or 100 µg/kg terazosin. Motor neurons were defined as cells in the ventral horn with the shortest diameter >20 µm, represented by white arrows. Scale bar=20 µm. (**D**) Quantification of number of motor neurons shows no significant difference in motor neuron number following terazosin treatment (t-test P=0.7428) N.s.=non-significant. Error bars represent S.E.M. Each point represents one mouse.

**Supplementary Figure 3. Terazosin treatment does not change glycolysis or mitochondrial respiration in ESC-MNs in unstressed conditions.** (**a**) Traces from the Seahorse analyser glycolytic stress assay showing glycolytic proton efflux rate (GlycoPER) following mitochondrial inhibition (oligomycin) and inhibition of the glycolysis pathway (2-deoxy-D-glucose; 2-DG). (**b**) ESC-MNs derived from TDP-43^M337V^ mice do not have a significantly different rate of basal glycolysis compared to TDP-43^WT^, and treatment with 2.5 µM terazosin (Tz) does not change basal glycolysis rates (One-way ANOVA P=0.03 with Tukey’s multiple comparison test: TDP-43^WT^ vs TDP-43^M337V^ P=0.576, TDP-43^WT^ vs TDP-43^M337V^ + 2.5 µM Tz P=0.18). (**c**) ESC-MNs derived from TDP-43^M337V^ mice do not have a significantly different rate of compensatory glycolysis compared to TDP-43^WT^, and treatment with 2.5 µM terazosin (Tz) does not change compensatory glycolysis rates (One-way ANOVA P=0.27 with Bonferroni’s multiple comparison test: TDP-43^WT^ vs TDP-43^M337V^ P>0.99, TDP-43^WT^ vs TDP-43^M337V^ + 2.5 µM Tz P=0.46). (**d**) Traces from the Seahorse analyser showing oxygen consumption rate (OCR) following mitochondrial inhibition (oligomycin), mitochondrial uncoupling (FCCP) and electron transport chain inhibition (antimycin A/rotenone). (**e**) There is no change in basal respiration between TDP-43^WT^-ESC-MNs, TDP-43^M337V^-ESC-MNs or TDP-43^M337V^-ESC-MNs (One-way ANOVA P=0.11 with Bonferroni’s multiple comparison test: TDP-43^WT^ vs TDP-43^M337V^ P>0.99, TDP-43^WT^ vs TDP-43^M337V^ + 2.5 µM Tz P=0.01). (**f**) There is no change in maximal respiration between TDP-43^WT^-ESC-MNs, TDP-43^M337V^-ESC-MNs or TDP-43^M337V^-ESC-MNs (One-way ANOVA P=0.53 with Bonferroni’s multiple comparison test: TDP-43^WT^ vs TDP-43^M337V^ p=0.56, TDP-43^WT^ vs TDP-43^M337V^ + 2.5 µM Tz P>0.99). (**g**) There is no change in ATP production between TDP-43^WT^-ESC-MNs, TDP-43^M337V^-ESC-MNs or TDP-43^M337V^-ESC-MNs (One-way ANOVA P=0.22 with Bonferroni’s multiple comparison test: TDP-43^WT^ vs TDP-43^M337V^ P>0.99, TDP-43^WT^ vs TDP-43^M337V^ + 2.5 µM Tz P=0.21). (**h**) There is no change in spare respiratory capacity between TDP-43^WT^-ESC-MNs, TDP-43^M337V^-ESC-MNs or TDP-43^M337V^-ESC-MNs (One-way ANOVA P=0.08 with Bonferroni’s multiple comparison test: TDP-43^WT^ vs TDP-43^M337V^ P=0.27, TDP-43^WT^ vs TDP-43^M337V^ + 2.5 µM Tz P=0.84). Each data point represents a separate differentiation; n=7 per glycolysis analysis; n=6 per respiration analysis. Error bars represent s.e.m., ns=non-significant
