# Supplementary material for: Targeting phosphoglycerate kinase 1 with terazosin improves motor neuron phenotypes in multiple models of amyotrophic lateral sclerosis
Source: eBioMedicine. 2022 Aug 11;83:104202. doi: 10.1016/j.ebiom.2022.104202 (PMC9482929; doi:10.1016/j.ebiom.2022.104202)
Supplement: Supplementary file 2 [file mmc2.docx]

**Movie S1.** Videos of touch-evoked escape response (TEER) test from uninjected control, C9orf72 MO and C9orf72 MO + PGK1 OE fish.

**Movie S2.** Videos of touch-evoked escape response (TEER) test from uninjected control, mutTDP-43 OE and mutTDP-43 OE + PGK1 OE fish.
