# Supplementary figures and images for: Targeting phosphoglycerate kinase 1 with terazosin improves motor neuron phenotypes in multiple models of amyotrophic lateral sclerosis
Source: eBioMedicine. 2022 Aug 11;83:104202. doi: 10.1016/j.ebiom.2022.104202 (PMC9482929; doi:10.1016/j.ebiom.2022.104202)

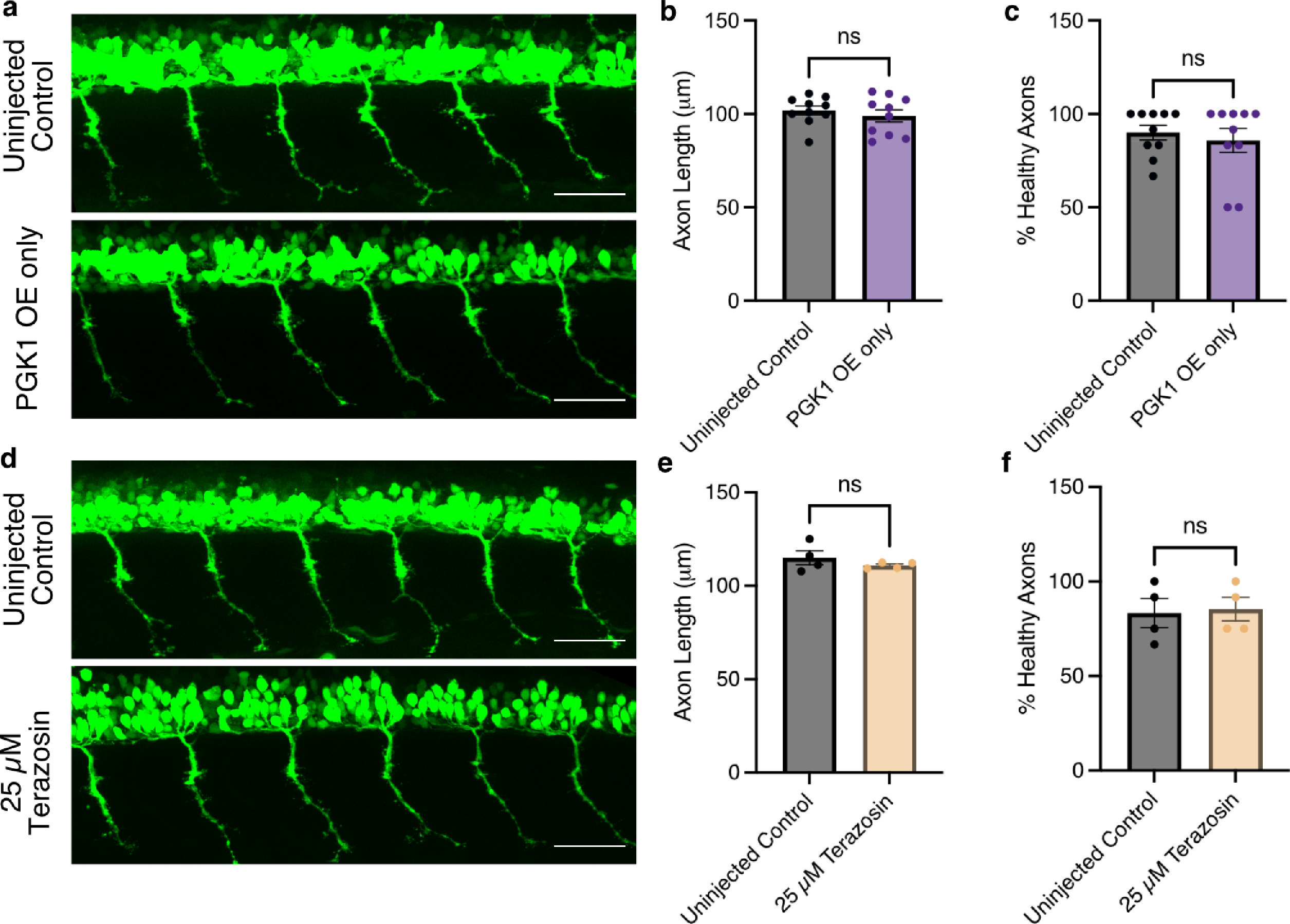

Supplement: Supplementary file 5 [file mmc5.jpg]

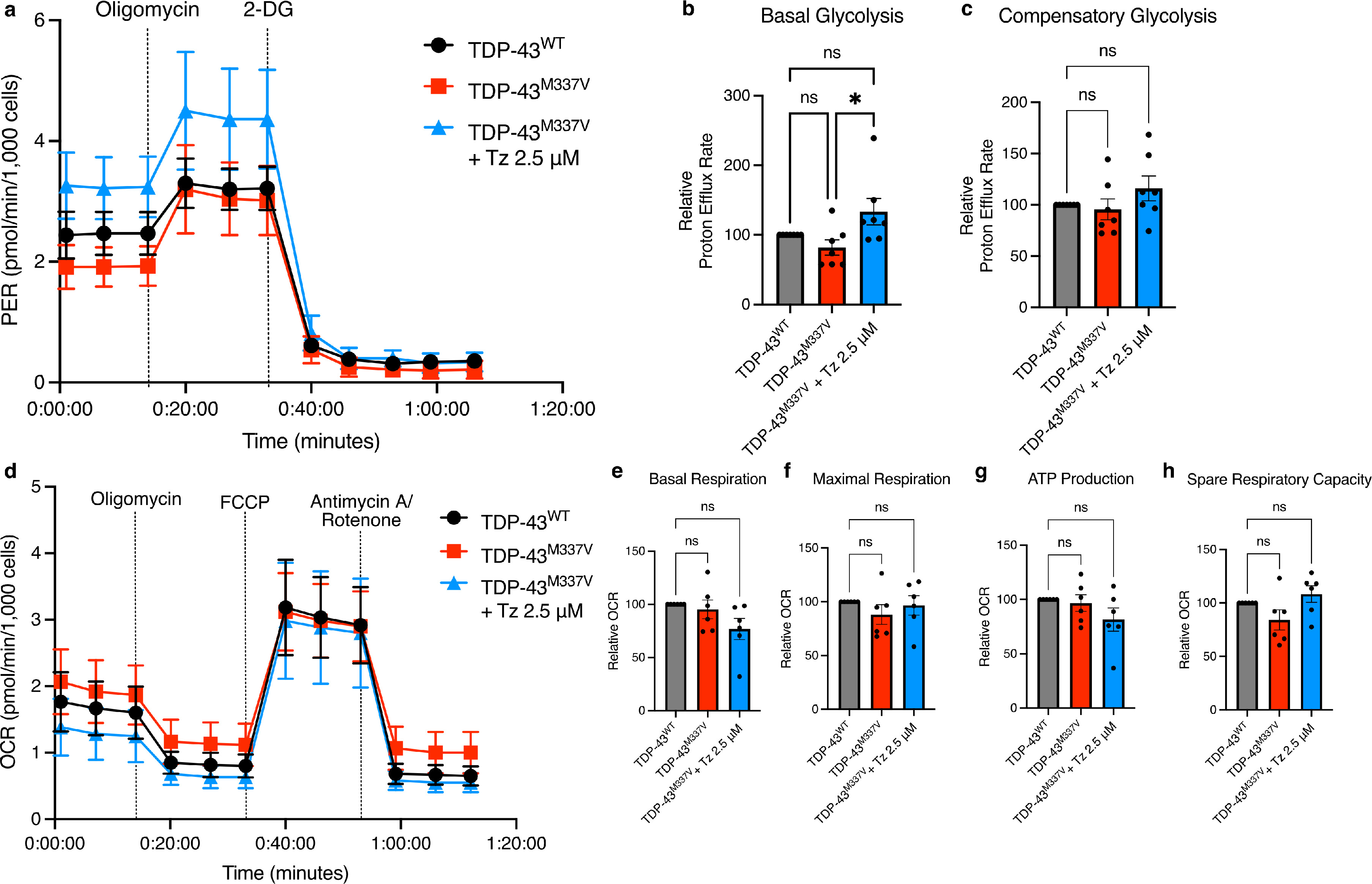

Supplement: Supplementary file 6 [file mmc6.jpg]

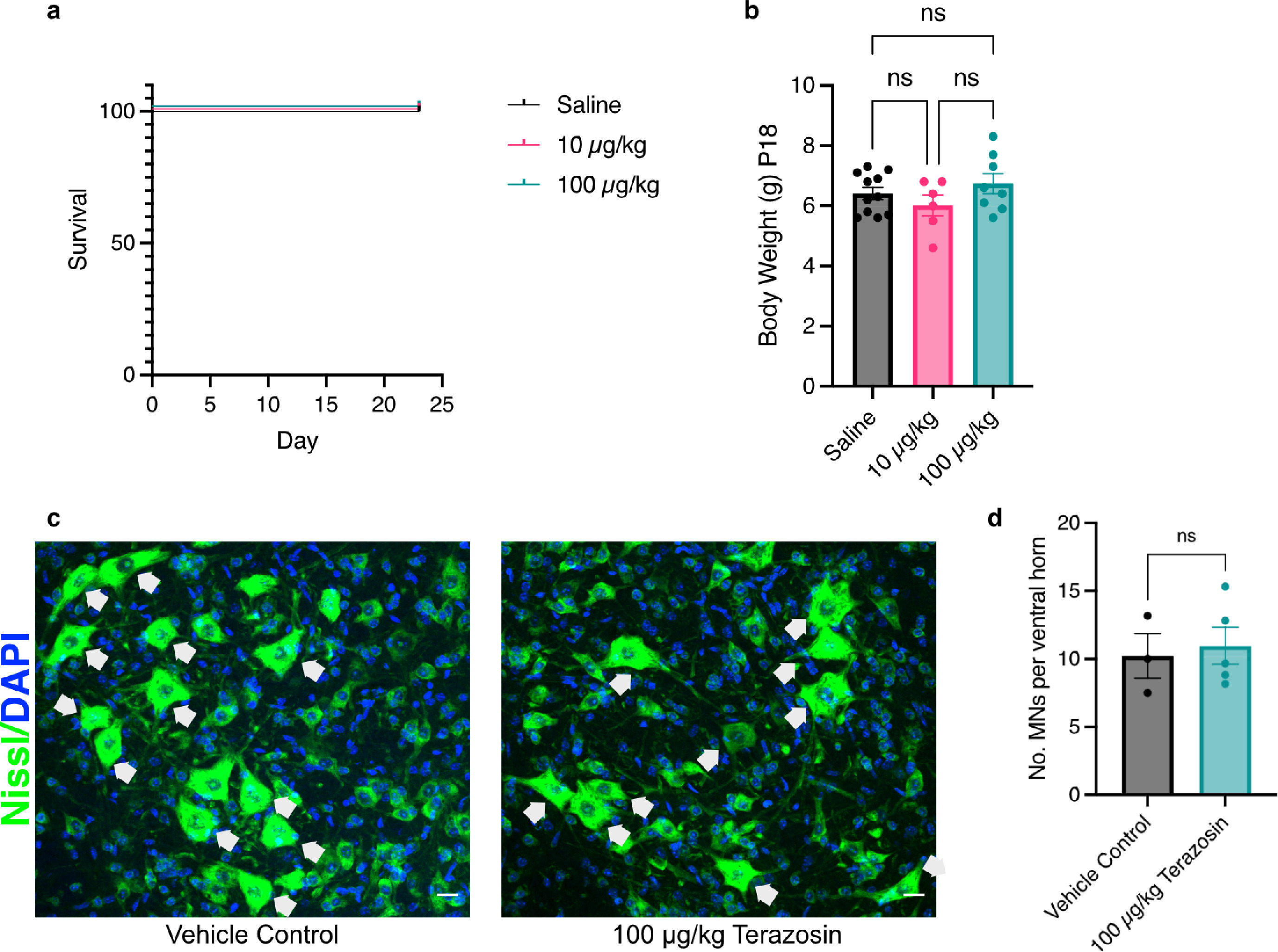

Supplement: Supplementary file 7 [file mmc7.jpg]
